# The Crystal Structure of the *Escherichia coli* RNase E Apoprotein and a Mechanism for RNA Degradation

**DOI:** 10.1016/j.str.2008.04.017

**Published:** 2008-08-06

**Authors:** Daniel J. Koslover, Anastasia J. Callaghan, Maria J. Marcaida, Elspeth F. Garman, Monika Martick, William G. Scott, Ben F. Luisi

**Affiliations:** 1 Department of Biochemistry, University of Cambridge, Tennis Court Road, Cambridge CB2 1GA, United Kingdom; 2 Department of Biochemistry, University of Oxford, South Parks Road, Oxford, OX1 3QU, United Kingdom; 3 Department of Chemistry and Biochemistry, and the Center for the Molecular Biology of RNA, Sinsheimer Laboratories, University of California at Santa Cruz, Santa Cruz, CA 95064, USA

**Keywords:** RNA, PROTEIN

## Abstract

RNase E is an essential bacterial endoribonuclease involved in the turnover of messenger RNA and the maturation of structured RNA precursors in *Escherichia coli*. Here, we present the crystal structure of the *E. coli* RNase E catalytic domain in the apo-state at 3.3 Å. This structure indicates that, upon catalytic activation, RNase E undergoes a marked conformational change characterized by the coupled movement of two RNA-binding domains to organize the active site. The structural data suggest a mechanism of RNA recognition and cleavage that explains the enzyme's preference for substrates possessing a 5′-monophosphate and accounts for the protective effect of a triphosphate cap for most transcripts. Internal flexibility within the quaternary structure is also observed, a finding that has implications for recognition of structured RNA substrates and for the mechanism of internal entry for a subset of substrates that are cleaved without 5′-end requirements.

## Introduction

RNase E is an essential endoribonuclease responsible for the degradation of most mRNA in *E. coli* (Mudd et al., 1990; Babitzke and Kushner, 1991). In addition to its purely degradative role, RNase E is necessary for the maturation of precursors of 5S ribosomal RNA (Apirion and Lassar, 1978; Misra and Apirion, 1979), 16S ribosomal RNA (Li et al., 1999), tRNAs (Ow and Kushner, 2002), and the M1 RNA component of the RNase P ribozyme (Lundberg and Altman, 1995; Ko et al., 2008). The activity of RNase E can also be targeted to defined transcripts in conjunction with small regulatory RNAs (Aiba, 2007; Vanderpool, 2007; Viegas et al., 2007). The 1061 residue enzyme is composed of two distinct functional regions. The N-terminal half forms the catalytic domain (residues 1–530), resembles its paralog RNase G, and is relatively conserved among prokaryotes (Marcaida et al., 2006). The C-terminal half has little intrinsic structure but serves as scaffolding for other enzymes and, in contrast to the N-terminal domain, is poorly conserved (Carpousis, 2007). RNA helicase B (RhlB), polynucleotide phosphorylase (PNPase), and enolase each bind to this scaffolding to form a large multiprotein complex known as the RNA degradosome (Marcaida et al., 2006; Carpousis, 2007). These components are proposed to act in concert to degrade and process cellular RNA.

**Figure 1:**
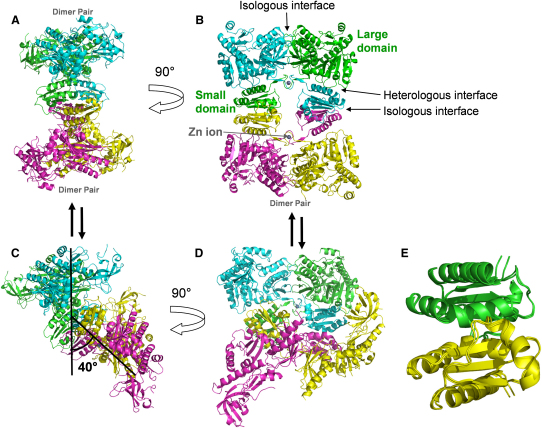
The Quaternary Organization of RNase E Is Flexible The protomers of the RNase E tetramer are colored pink, yellow, green, or cyan. The pink and yellow protomers form a dimer pair, as do the green and cyan protomers (A). The RNase E tetramer is observed to have D_2_ symmetry in the previously reported holoprotein configuration (A and B) and here, a large kink of about 40° was observed in the apoprotein structure (C and D). The isologous and heterologous domain-domain interfaces are indicated (B). The zinc ions are shown in gray. Quaternary structural changes are restricted to the heterologous interfaces. The bend in each tetramer was calculated by superimposing the RNase H and DNase I regions of a single dimer from each model and calculating the resulting angle between zinc ions. The dimer-dimer interaction mediated by the small domains is most easily viewed as in panel B, and we have labeled the large and small domains of an individual protomer (green in [B]) for clarity. It is clear that the dimer-dimer interaction is virtually identical in each structure when the small domains from each are superimposed (E). We found that the observed change in quaternary organization is due to a change in the orientation of the small domains relative to the large, but that in each case the region coordinating a zinc ion is unaltered.

**Figure 2:**
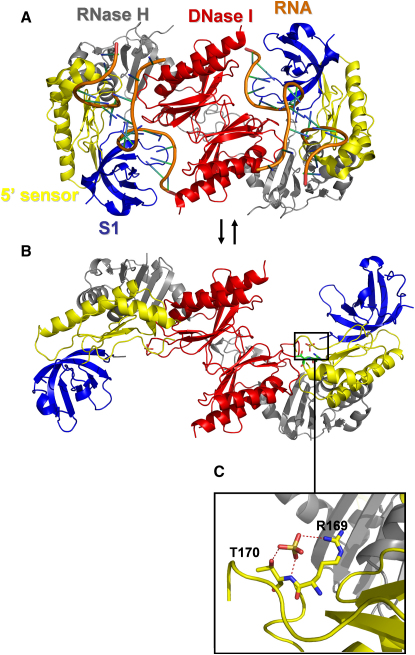
Holoprotein and Apoprotein States of the RNase E Tetramer The view from above relative to the orientation in Figure 1B and only a dimer pair is shown for clarity. The protomers have been colored according to subdomain structure. The large conformational change of the consolidated 5/S1 subdomain (residues 36–214; blue and yellow) between the closed (A) and open (B) states is most evident when the two structures are juxtaposed and viewed along the preserved dyad symmetry element of the dimer formed by the DNase I-like subdomains. In the apoprotein structure, a putative sulfate group (C) is hydrogen-bonded to R169 and T170 in the same manner as the 5′ monophosphate group in the holoprotein within the 5′ sensing pocket.

The crystal structures of the *E. coli* RNase E catalytic domain, bound to 10-mer, 13-mer, and 15-mer 2′-O-methyl-protected RNA substrates, were recently solved by X-ray crystallography to 2.9 Å resolution (Figures 1A and 1B; Callaghan et al., 2005a). These structures reveal a closed conformation in which the protein is clamped down on the RNA substrate. The RNase E catalytic domain forms a dimer of dimers, with a quaternary organization resembling two pairs of scissors arranged in tandem. Each protomer possesses one large and one small domain on either side of the scissor junction point composed of residues 1–400 and 415–510, respectively. Between these two domains is a pair of conserved CPxCxGxG motifs, one on each monomer, that coordinate a single zinc ion. Mutation or deletion of this binding motif prevents tetramer formation and substantially reduces RNase E catalytic activity (Callaghan et al., 2005b; Caruthers et al., 2006). Each large domain can be divided into four subdomains on the basis of function and similarity to homologous structural folds (Figure 2A). Residues 1–35 and 215–279 together form the RNase H subdomain, which is structurally similar to the RNase H endoribonuclease but lacks the critical catalytic residues (Callaghan et al., 2005a). Likewise, the DNase I subdomain is named for its structural similarity to an established fold found in an endonuclease that has specificity for duplex DNA. The self-complementary interactions of the DNase I subdomain (residues 280–400) dominate the dimer interface in RNase E. The S1 subdomain (residues 36–118) and 5′ sensing region (residues 119–214) are embedded within the RNase H subdomain and appear to be critical for binding and orienting substrate RNA for cleavage; we elaborate more on this later.

A distinctive feature of RNase E/G is the preference for RNA substrates with a free 5′ terminus and the ability to cleave RNA at a distance from the 5′ end. This has been demonstrated by the striking finding that circularization of an RNA substrate will substantially decrease its cleavage rate by RNase E (Mackie, 1998, 2000). Endonucleolytic cleavage is also impeded by base-pairing at the 5′ end of the RNA (Mackie, 2000; Bouvet and Belasco, 1992). Catalytic rates are greater for substrates with a 5′-monophosphate versus those with a free hydroxyl group or triphosphate cap (Mackie, 1998; Jiang and Belasco, 2004). The previously reported crystal structure of the *E.coli* RNase E catalytic domain (Callaghan et al., 2005a) reveals that the 5′-monophosphate on the substrate is bound in a pocket on the ribonuclease and that recognition is mediated through hydrogen bonding between the phosphate groups with the main-chain amide of T170, as well as the side chains of T170 and R169. This 5′ sensing site is located at a distance from the catalytic site, which resides on the DNase I subdomain. There, D303 and D346 coordinate a magnesium ion that likely mediates cleavage by hydrolytic attack of the RNA backbone. A shallow hydrophobic pocket located on the S1 subdomain interacts with one of the RNA bases and helps to orient the substrate in the catalytic site. The catalytic and 5′ sensing sites are positioned by the quaternary structure such that RNA is bound by one monomer but cleaved by its partner within the dimer structure.

Here, we present the first crystal structure of the *E. coli* RNase E catalytic domain in the apo form at 3.3 Å resolution. We find a striking conformational change in which the 5′ sensor and S1 subdomains move as a single unit through an angle of ∼60° between the apoprotein and holoprotein states (Figure 2B). This conformational change suggests a mechanism of RNA recognition and catalysis that explains the enzyme's preference for substrates possessing a 5′-monophosphate over triphosphate and hydroxy-capped RNA. We propose that triphosphate caps or secondary structure in the terminus of the transcript protect the RNA against degradation by directly impeding the conformational change required for catalysis in RNase E, thus preventing premature turnover of transcripts. We also observe substantial flexibility of the quaternary structure, as indicated by a bending at one of the dimer-dimer interfaces, a deformation that may be required to accommodate structured RNA for processing by internal entry.

## Results and Discussion

### Crystallographic Diffraction Data

Crystallographic data and model statistics for the apoprotein are presented in Table 1. A second structure possessing small fragments of M1 RNA bound to RNase E and possessing a tertiary structure nearly identical to that of the apoprotein was solved at 3.5 Å, and diffraction data for this structure are presented in the Supplemental Data available online. The positions of the side chains of several residues in both structures are not certain because of the poor quality of the electron density maps. However, the models can be interpreted more confidently in regard to subunit and subdomain organization, to which we now turn.

### Quaternary Organization of RNase E

The quaternary structure of the RNase E apoprotein is composed of a dimer-of-dimers connected via the small domains but has substantial deviations from the D_2_ symmetry observed in the holoprotein (Figures 1A–1D). Although the small domains form self-complementary dimer interfaces that are identical to those previously observed (Figures 1B and 1E; Callaghan et al., 2005a), these interfaces are reoriented relative to the large domains. Specifically, each is twisted by about 45° in the apoprotein relative to its orientation in the holoprotein, and the tetramer is bent out of the plane by ∼40° (Figures 1C and 1D). Although the twisting and bending has affected the overall shape of the whole zinc-coordinating region (residues 401–414), the structure of the zinc-binding pocket itself (residues 404–407) does not significantly change. Two zinc ions are present in the structure, one in each pocket.

The observation that the quaternary structure is bent implies that the RNase E tetramer must possess a relatively large degree of flexibility about the dimer interface formed between the small- and large-domains. We refer to this interface as a “heterologous” domain-domain interface since it involves contacts made by different types of subdomains (Figure 1B). In contrast, there is little change at the “isologous” interfaces that are formed by contacts between the same types of subdomains, such as the interfaces between the large domains that are mediated by its paired DNase I-like subdomains, or the self-complementary interfaces of the small domains. This finding is perhaps not unexpected given the hydrophobic nature of both the DNase I/DNase I and small domain/small domain isologous interfaces. The apoprotein and holoprotein crystals were grown in different crystallographic space groups, under different conditions, and with different lattice contacts, so it seems that the barrier for conformational change at the “heterologous” large/small domain interface is on the order of the crystal lattice packing energies. We hesitate to assign any significance to the particular quaternary organization observed in the crystal structure; instead, we consider that the changes observed here reflect the flexibility of the quaternary structure. A flexible quaternary structure for the apoform of RNase E is in accord with small-angle X-ray and neutron solution-scattering profiles (Grossmann et al., 2008). Quaternary flexibility is also suggested by a second RNase E structure that we have solved to 3.5 Å and that possesses small breakdown fragments of M1 RNA (Supplemental Data). In that structure, the quaternary organization resembles the previously described holoprotein, although possessing a slight ∼10° bend, and its tertiary organization is nearly identical to that of the apoprotein (Figure S1).

In vivo and in vitro studies suggest that tetramer formation is necessary for full RNase E functionality (Callaghan et al., 2005b; Caruthers et al., 2006), but there is currently no known structural basis to account for these observations. One possible function of the quaternary structure may be to process long substrates with positive or negative cooperativity, which could be achieved through communication between subunits mediated through heterologous and isologous domain-domain interactions. We have not observed any apparent cooperativity for small substrates, such as 13-mers, but it is possible that cooperative effects might be seen in the cutting of larger substrates such as mRNA. A second role for the tetramer may be the accommodation of structured RNA precursors or mRNAs that are cleaved by internal entry (Hankins et al., 2007; Baker and Mackie, 2003; Joyce and Dreyfus, 1998).

Quaternary structural adjustments may occur in the binding of intricately folded RNAs, such as M1 RNA. A complex of RNase E with such a large, structured RNA would have two or more equivalent binding sites and would be expected to have a ratio of one RNase E tetramer with two or more RNA if it were to maintain perfect symmetry. However, the complex between the N-terminal catalytic half of RNase E and M1 RNA has one tetramer per RNA component under saturating conditions, as shown by native gel electrophoresis mobility shift assays and by nondissociating mass spectrometry (P. Ilag et al., personal communication). A bent tetramer may explain this observed 1:1 ratio of stoichiometry of tetramer to RNA. Further experiments are needed to determine the role of RNase E tetramer organization in the maturation of structured RNA.

### Subdomain Reorganization of RNase E Protomers with RNA Binding

In each of the four apoprotein protomers, the combined S1 subdomain and 5′ sensor (from here on referred to collectively as the “5/S1 subdomain”) has moved as a single unit through an angle of about 60° relative to the holoprotein configuration (Figure 2B). This conformational change significantly exposes both the binding and catalytic sites to the surrounding solvent and probably permits substrates to be more easily bound to the enzyme. Additional electron density was identified in the 5′ sensing pocket in three of the four monomers. On the basis of the coordination geometry and the buffer composition, we propose that this density is likely to be a sulfate ion. This ion is hydrogen-bonded to the T170 side chain and amide group, as well as to the R169 side chain, mimicking the interaction between RNase E and the 5′-monophosphate group of RNA seen in the holoprotein structure (Figure 2C). The R169 side chain also interacts with the main-chain carbonyl oxygen of G124, a residue previously implicated as having a role in orienting R169 in the 5′ sensing site.

In their report of the holoprotein structure of RNase E, Callaghan et al. (2005a) speculated that a change in the position of the S1 subdomain represents the major difference between the apoprotein and holoprotein states. The structures presented here corroborate this movement, but the magnitude of the conformational change between the open and closed states is much greater than expected. Also unanticipated was the movement of the S1 and 5′ sensor domains as a single body. In the open configuration, the 5′ sensing and catalytic sites are highly exposed to the surrounding solvent, suggesting that RNase E can easily bind large RNA molecules, with potential implications for recognition of large RNA substrates that have complex secondary structure.

The holoprotein model identified a magnesium ion in the catalytic site coordinated by D303 and D346 with the support of N305. Each of these residues has been implicated from mutation studies as required for catalytic activity (Callaghan et al., 2005a). In the structure presented here, these residues are oriented as in the previously reported holoprotein, but no electron density was apparent near the catalytic sites, suggesting that the metal may be absent. It is possible that the magnesium ion is corecruited with RNA during ligand binding.

### A Mechanism of Substrate Degradation by RNase E

**Figure 3:**
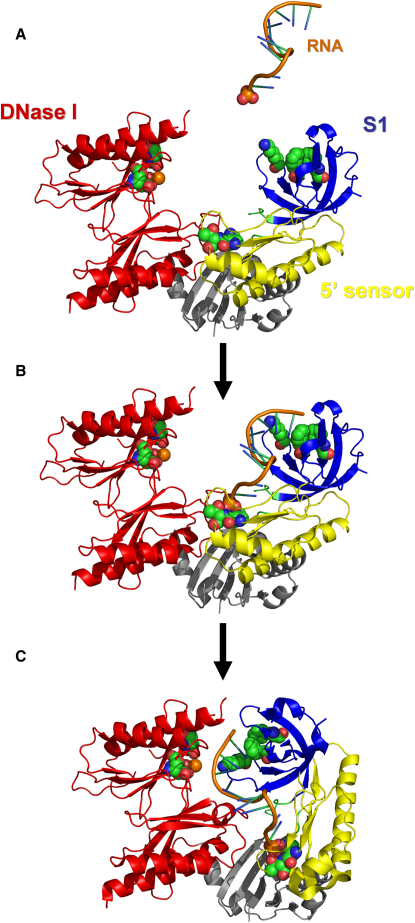
Proposed Mechanism of Substrate Binding and Catalysis by RNase E (A) In the absence of RNA, the monomer is in an open state in the highly dynamic apoprotein state. The S1 subdomain and 5′ sensing site are both exposed to the surrounding solvent, allowing RNA to readily bind. (B) The 5′ sensing pocket likely contributes a significant portion of the substrate-binding affinity, with the S1 subdomain acting to orient the molecule. (C) After RNA is bound, the consolidated 5/S1 subdomain moves as a unit in a conformation change that brings the substrate into close proximity to the catalytic site on the DNase I subdomain. The RNA is cleaved and the products are released as the structure returns to the open configuration. Depicted in space-filling representation are the key recognition residues of the 5′ sensing pocket and the S1 subdomain, the catalytic residues on the DNase I domain, and the 5′ phosphate of the single-stranded RNA substrate.

Callaghan et al. (2005a) proposed that binding of RNA to the 5′-monophosphate pocket triggers the movement of the S1 subdomain in an “induced fit” mechanism. We propose a revised mechanism of RNA recognition and degradation by RNase E based on the structure presented here, in which the S1 and 5′-sensor together form the main allosteric body. A summary of the key steps proposed by the model is illustrated in Figure 3. First, RNA binds to the combined S1 subdomain and 5′ sensor in the open configuration. The RNA is anchored primarily by the binding affinity of the 5′ sensor (R169 and T170) and oriented by the hydrophobic surface patch on the S1 subdomain. These two sites hold the RNA while the consolidated 5/S1 subdomain moves as a single unit into the closed configuration. This brings the substrate into close proximity to the catalytic site where a nucleophilic attack on the phosphate backbone by a hydroxyl group is mediated by a magnesium ion. The RNA is cleaved, and the reaction products are subsequently released as RNase E returns to the open configuration.

We favor this model because it accounts for the preference of the enzyme for substrates with a 5′-monophosphate terminus over those with either a triphosphate or hydroxy cap. It predicts that with only a terminal hydroxy cap, binding of an RNA substrate by RNase E will be substantially weaker and cleavage will be impeded under nonsaturating conditions. If a triphosphate cap is present on substrates, then the 5′ sensing site may still be able to bind them with moderate affinity because there is sufficient space in the open configuration of the enzyme to accommodate the three phosphate groups. However, RNA cleavage will again be greatly impeded because the extra phosphates are likely to sterically clash with the rest of the structure during the transformation to the closed configuration, acting as a wedge at the base of the fulcrum. Furthermore, the movement of these charged groups into a hydrophobic environment represents an additional thermodynamic barrier to domain closure.

Our crystal structure suggests that the enzyme will bind isolated phosphate and sulfate groups within the 5′ terminal recognition site. Thus, it seems likely that the recognition site residues make a key contribution to the RNA binding affinity. In this respect, our model conflicts with reports that the 5′ end of the substrate provides no preference for RNA binding affinity and that the effect of the 5′-monophosphate is primarily due to its role in catalytic activation (Jiang and Belasco, 2004). However, our results are consistent with a more recent binding study indicating that 5′ terminal recognition site residues in the RNase G homolog are indeed responsible for significant binding affinity (Jourdan and McDowall, 2008).

Although the model for substrate interaction effectively describes global, nonspecific RNA degradation by RNase E, the mechanism used to process complex substrates through restricted cleavage at only specific sites is still unknown. The open configuration of the apoprotein may allow these more complex RNAs to be accommodated within the active site and bound at the 5′ end without the requirement for the same structural change that maneuvers small single-stranded RNA into the cleavage orientation. We would envisage that a single-stranded segment would be accommodated into the shallow channel that leads to the active site. The secondary structure of complex substrates may be sufficient to bring a defined RNA segment into close proximity to the catalytic site. In such cases, the substrates may not depend on RNase E undergoing a conformational change and may be effectively cleaved in the presence of either a 5′ mono or triphosphate cap. Likewise, it is also possible that the observed open configuration may allow a complex substrate to bind to a single protomer while positioning another segment of the RNA to be cleaved by a second protomer. Such a model would help to explain the functional value of the enzyme's tetrameric organization and is particularly attractive because of the observed flexibility of the structure.

A recent report indicates that the conversion of a 5′-triphosphate cap to a monophosphate represents the rate-limiting step in bacterial mRNA decay (Celesnik et al., 2007). Recent biochemical studies have identified a phosphatase that catalyzes 5′ pyrophosphate removal from transcripts so that they become favored substrates for RNase E (Deana et al., 2008). On the basis of our structural models, we suggest that a triphosphate cap protects transcripts from premature destruction by directly impeding the conformational change required for cleavage by RNase E. The removal of phosphate groups from a 5′ terminus therefore represents the critical step that marks mRNA for destruction, implying that the preference of RNase E for substrates possessing a 5′-monophosphate is fundamental to proper bacterial gene regulation and transcript turnover.

## Experimental Procedures

### Solution of Crystal Structures by Molecular Replacement

The crystallization of the RNase E apoprotein and subsequent data collection have previously been described (Callaghan et al., 2003). Data were processed using Denzo and were scaled with Scalepack (Otwinowski and Minor, 1997). The CCP4 suite programs PHASER (Storoni et al., 2004) and MOLREP (Vagin and Teplyakov, 1997) were used to construct an initial RNase E apoprotein model using molecular replacement. Individual protein domains from the existing holoprotein tetramer (PDB entry: 2BX2) (Callaghan et al., 2005a) were used as search models to iteratively build the structure. The DNase I and RNase H domains were placed first within the tetramer using PHASER, followed by the 5′ sensor domains, small domains, and S1 domains using a combination of PHASER, MOLREP, and direct placement based on tetramer symmetry. This model was refined using MOLREP rigid-body refinement, followed by manual building with Coot (Emsley and Cowtan, 2004) and multiple iterative cycles of translation-libration-screw (TLS) displacement combined with restrained refinement, unrestrained refinement, and structure idealization in Refmac (Winn et al., 2001). The “Zn-link” domains were built into the electron density manually, and the positions of the zinc ions were independently corroborated by an anomalous Fourier synthesis (the diffraction data for the apo form were collected at the zinc edge, 1.28 Å). Although this resulted in a model of the tetramer with R_free_ ∼28.5%, the Ramachandran plot and side-chain electron density were poor and suggested that the data were overfitted. Thus, a new model was constructed by overlaying the protein domains from the holoprotein structure (DNase I, RNase H, combined 5′ sensor + S1 domain, and the small domain) over their equivalents in the first apoprotein model, running MOLREP rigid-body refinement on these, and manual rebuilding using Coot. The model was then refined again using TLS plus restrained refinement and structure idealization in Refmac. The positions of the zinc ions were again confirmed by an anomalous Fourier synthesis. This later model has electron density and stereochemistry significantly improved over the first and is presented here. The structure was examined and validated using PROCHECK (Laskowski et al., 1993) and SFCHECK (Vaguine et al., 1999) and was shown to have appropriate chemical bond lengths, phi-psi backbone angles, and chi angles. No residue possesses disallowed Ramachandran geometry. Structural figures were prepared using PyMOL (Delano, 2002).

## Figures and Tables

**Table 1:** Diffraction Data and Refinement Statistics

	RNase E Apoprotein
**Diffraction Data**	
Space group	P1
Unit cell dimensions	a = 73.24, b = 75.57, c = 109.37 Å
	α = 94.95, β = 102.03, γ = 91.77°
Resolution (Å)	30.0−3.3 (3.42−3.30)
Number of unique reflections	32,518 (3,144)
Multiplicity	3.8 (3.2)
Completeness (%)	98.1 (94.2)
I/σ	11.8 (2.3)
R_merge_ (%)	10.8 (44.7)
Wilson B factor (Å^2^)	70.6
**Refinement Statistics**	
Resolution (Å)	25.0−3.3
R factor	0.272
R_free_	0.294
Number reflections used	33,032
Total number of atoms	14,540
Total number of amino acid residues	1,954

Crystallographic statistics were calculated by use of Scalepack (Otwinowski and Minor, 1997) and SFCHECK (Vaguine et al., 1999). Refinement statistics were calculated by use of Refmac (Winn et al., 2001). All resolution shells were used for refinement of the apoprotein. No Ramachandran outliers are present.

## Accession Numbers

The coordinates and structure factors for the RNase E apo and RNase E/M1 fragment structures have been deposited with the protein structural databank with accession numbers 2VMK and R2VMRSF (apo form) and 2VRT and R2VRTSF (M1-fragment form).

## Supplemental Data

Document S1. Two Figures, One Table, Supplemental Experimental Procedures, and Supplemental References
