# Supplementary material for: The Crystal Structure of the Escherichia coli RNase E Apoprotein and a Mechanism for RNA Degradation
Source: Structure. 2008 Aug 6;16(8-2):1238–44. doi: 10.1016/j.str.2008.04.017 (PMC2631609; doi:10.1016/j.str.2008.04.017)
Supplement: Document S1. Two Figures, One Table, Supplemental Experimental Procedures, and Supplemental References [file mmc1.pdf]

Structure *16*

Supplemental Data

The Crystal Structure of the *Escherichia coli* RNase E Apoprotein and a Mechanism for RNA Degradation

Daniel J. Koslover, Anastasia J. Callaghan, Maria J. Marcaida, Elspeth F. Garman, Monika Martick, William G. Scott, and Ben F. Luisi

Supplemental Experimental Procedures

RNase E Complexed with Small Fragments of RNase P at 3.5 Å

Crystallographic data and model statistics for RNase E bound to nucleotides of M1 RNA are presented in Table S1. Although crystallizations of the complex were carried out with the addition of either a 377 or 327 nucleotide derivative, the crystals possess only a few nucleotides. An elemental analysis of the crystals by microPIXE (Proton Induced X-ray emission spectroscopy) (Garman and Grime, 2005) places an upper limit of ~40 phosphates per tetramer (see below for methods). We could identify ten nucleotides in the structure. Additional bases of RNA may be present, but these could not be accounted for given the resolution and the presence of disordered regions. Two of the four S1 sub-domains could not be built as well as one of the zinc-coordinating peptides.

The structure of the M1-fragment/RNase E complex is in many respects intermediate between that of the holo-form (Callaghan *et al.*, 2005) and the apo-form described in the main text. Although the quaternary organization is deformed relative to the D_2 symmetry of the original holo-form, it is still nearly planar and only possesses a slight ~10° bend as compared to the much greater ~40° bend of the apo-form

(Figures S1A and S1B). Aside from the differences in the heterologous domain-domain interactions that produce this bending of the tetramer, the tertiary organization of the individual protomers is nearly identical to that of the apoprotein structure.

A short strand of two to three nucleotides is seen in each protomer within the structure (Figure S2). Just as the putative sulfates in the apoprotein are hydrogen-bonded to residues R169 and T170, so is the 5'-monophosphate group for each strand. Relative to the consolidated 5/S1 sub-domain, the first two nucleotides are oriented identically to those found at the 5' end of the previously solved holoprotein structures. Where present, the third nucleotide base of M1 RNA binds to a hydrophobic surface patch on the S1 sub-domain (residues F57 and F67) of an adjacent tetramer (Figure S2B). There, the base is rotated by about 90° relative to the holoprotein substrate, but in the same plane as the nucleotide rings. Thus, the purine rings remain parallel to the side chain of F67 and the π-π stacking interaction is maintained between the two structures.

RNase E – M1 RNA Complex Crystallization, Data Collection, and Processing

For the co-crystallisation of RNase E catalytic domain to fragments of M1 RNA, a truncated form of M1 RNA lacking bases 156-205 and with total length 377 or 327 nucleotides was mixed with RNase E in a roughly 1:4 RNA to protein ratio and incubated for 30 minutes. The complex was concentrated and crystals grown using the sitting drop vapor diffusion method in Hampton Membfac screen condition number 34 (10% PEG 4000, 100 mM $(NH_4)_2SO_4$, and 100 mM HEPES pH 7.5). A single crystal was serially transferred into 10, 15, 20, 25, and 30% v/v

ethylene glycol cryoprotectant before rapid cooling in liquid nitrogen. Diffraction data at wavelength 0.975 Å were collected at station ID29, ESRF, Grenoble, France. These data were processed using Mosflm (Leslie, 1992) and scaled with Scala (CCP4, 1994).

Solution of RNase E – M1 RNA Complex Structure

PHASER and MOLREP were used to construct a model of RNase E within the crystal by molecular replacement. As in the apoprotein, individual protein domains from the existing holoprotein tetramer (PDB entry 2BX2) were used as search models to iteratively build the structure. The DNase I domains were placed first within the tetramer using PHASER, followed by two RNase H domains, two 5/S1 domains, a small domain, the remaining RNase H domains, and the remaining 5' sensors. The second small domain was placed using a MOLREP search within the difference map of the structure and the third small domain found using PHASER. The fourth small domain was added directly by hand, based on the relative positions of the small domains already present. The model was refined using MOLREP rigid-body refinement, and both the Zn-link domains and initial RNA fragments were added in Coot based on homology to the holoprotein structure. Missing residues were then added to the structure using Coot to the extent possible from the electron density map. The structure was refined using TLS combined with restrained refinement and structure idealization was carried out in Refmac. The structure was examined and validated using PROCHECK (Laskowski *et al.*, 1993) and SFCHECK (Vagin *et al.*, 1999), and shown to have appropriate chemical bond lengths, phi-psi backbone angles, and chi angles. No residues have disallowed Ramachandran geometry.

MicroPIXE

Data were collected at the Surrey Ion Beam Centre (University of Surrey). A 2.5 MeV proton beam of 2 μm diameter was used to induce characteristic X-ray emission from protein samples under vacuum. The sample to detector distance was 25 mm. The X-rays were detected in a solid-state lithium-drifted silicon detector with high energy resolution, placed at an incident angle of 45° with respect to the incident beam. The crystals examined were RNase E catalytic domain with RNase P M1. RNase P M1 contains 377 nucleotides, i.e. 377 phosphorus atoms. From the crystal structure of RNase E catalytic domain, it is known that there is 1 zinc atom per RNase E dimer. The protein buffer was 20 mM Tris, 500 mM NaCl, 0.5 mM EDTA, 10 mM $MgCl_2$, 10 mM DTT and glycerol. RNase P was stored in water. The crystals were grown in 10% PEG 4K, 100 mM $(NH_4)_2SO_4$, and 100 mM HEPES pH 7.5. The crystal of the RNase E catalytic domain/15-mer single-stranded RNA complex for which the structure has been determined, were used as a control. The 15-mer RNA contains 15 phosphorus atoms, and there is one molecule of RNA per RNase E protomer. One zinc is expected per dimer. The protein buffer was 20 mM Tris, 500 mM NaCl, 0.5 mM EDTA, 10 mM $MgCl_2$ and 10mM DTT. These crystals were grown in 100 mM Tris pH 7.8, 20 mM Mg formate 2% PEG 8000 and 0.1 mM Na azide. Normally the ratio of sulphur to phosphorus signal would be used to determine the RNA species present per protein molecule. However, in this case there is extra sulphur in each sample from corresponding buffers that would interfere with the sulphur signal from the methionine and cysteine residues. Therefore the Zn/P ratio was measured. This assumes that in the RNase E/RNase P complex crystals, RNase E binds the same number of Zn atoms per protein molecule as the control RNase E/15-mer complex. One

RNase E/15-mer RNA complex crystal and two RNase E/RNase P complex crystals were prepared for the experiment. They were transferred from the crystallization drop to deionized water and washed three times in order to remove the buffer. The crystals were then transferred onto 2μm thick mylar film sample holders (Garman and Grime, 2005). The samples were aligned in the beam and a broad two dimensional scan (1 mm x 1 mm) was performed to localize the crystal to be examined. After location of the crystal, a 200 x 200 μm scan was performed. In order to measure element concentrations, point spectra were collected at three selected points in the crystal and also at a point on the backing foil at different positions in each of the crystals for 10 min. The spectra were analysed by the DAN32 and GUPIX software (Grime, 2004, Campbell et al., 2000).

Analysis of control crystals of RNase E/15-mer RNA had an average of 0.63 $\pm$ 0.10 Zn/protein molecule, reasonably consistent with the known result (0.5 Zn/protein molecule). The standard deviation on the 3 measurements was only 0.04, but this does not take into account systematic errors to do with the X-ray cross section databases. RNase E/RNase P M1 complex crystals had, on average, 0.065 Zn/P atoms. If we assume there are 0.63 Zn atoms per RNase E monomer, this implies that there are only 10 phosphorus bases/RNase monomer bound in the crystal. There was also a signal detected from the element Cu. The calculations obtained a stoichiometry of 0.030 Cu/P, which would be equivalent of one Cu per two Zn atoms, or one Cu per RNase E tetramer.

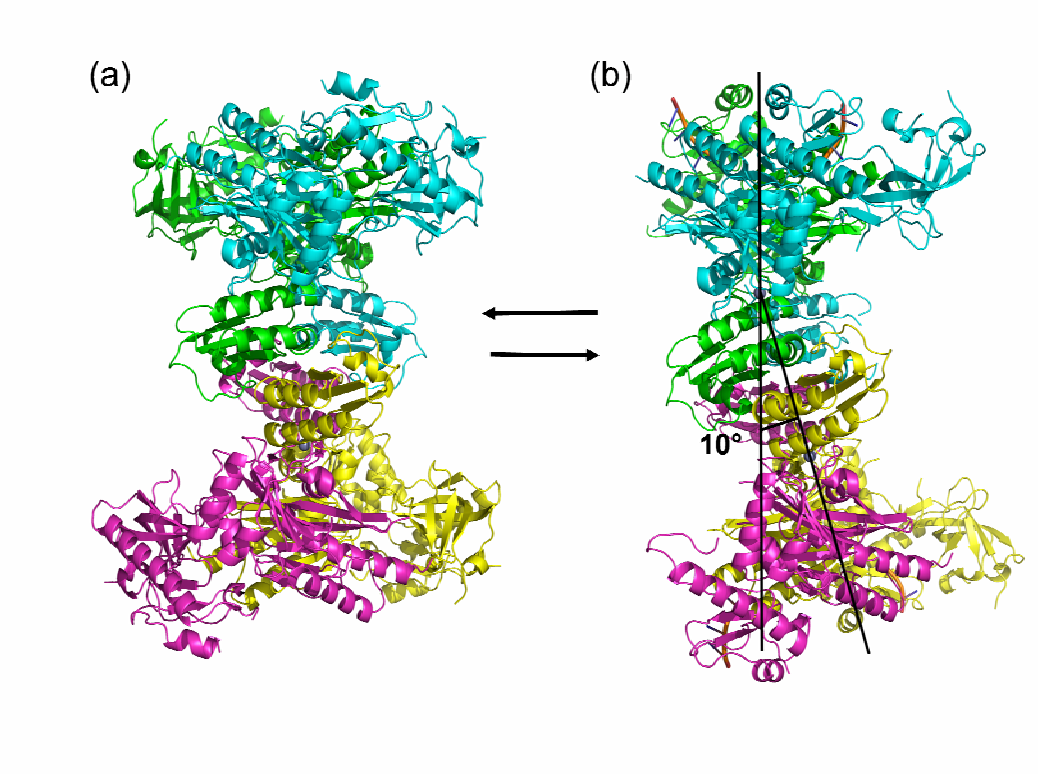

Figure S1. A 10° Bend in the Quaternary Organization of RNase E Is Apparent When Comparing the Original Holo-Form (a) to the M1-Fragment/RNase E Complex (b)

This conformational change is increased substantially in the apoform presented in the main text.

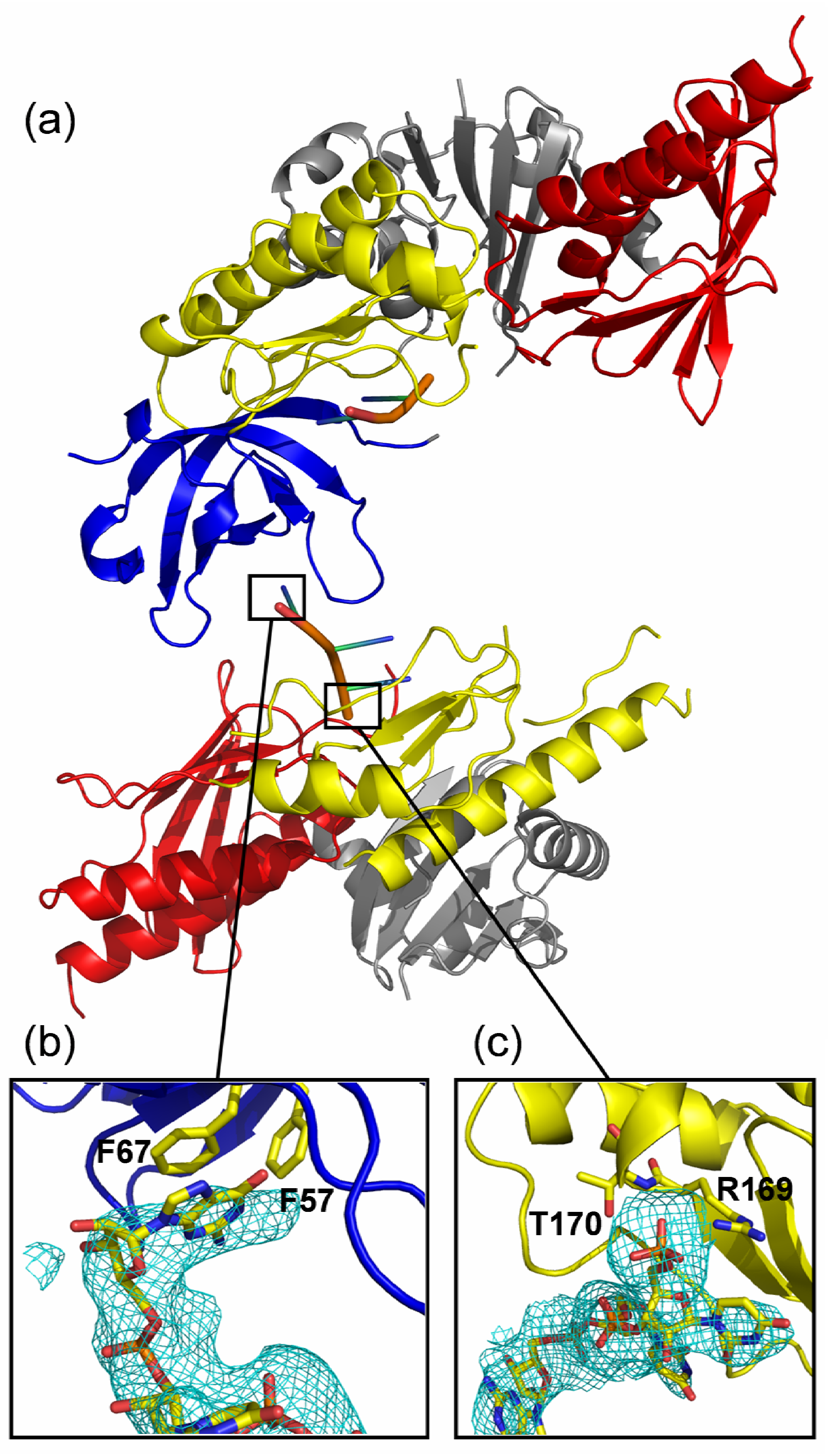
(a)
(b)
(c)
F67
F57
T170
R169

Figure S2. M1 RNA Fragments Connect Adjacent Tetramers within the Crystal Structure

Shown here are (a) two large domains in adjacent unit cells within the RNase E – M1 RNA crystal structure, only one of which (top) possesses an ordered S1 domain. A three-nucleotide fragment of RNA stretches between these two monomers and interacts with the hydrophobic surface patch on the S1 sub-domain at one end (b) and the 5’ sensing pocket at the opposite terminus (c). The electron density shown represents a simulated annealing omit map for the RNA, generated using PHENIX starting from the refined model in which the RNA was removed.

Table S1. Diffraction Data and Refinement Statistics

	**RNase E – M1 RNA fragment**
**Diffraction data**	
Space group	C2
Unit cell dimensions	a = 93.89, b = 121.25, c = 242.23 Å<br>α = 90.00, β = 99.47, γ = 90.00°
Resolution	79.5 - 3.50 Å
Number of unique reflections	33891
Completeness	99.9%
I/sigma	9.6 (3.3)
Rmerge	12.3 (39.2) %
Wilson B factor	57.9 $Å^2$
**Refinement statistics**	
Resolution	25.0-3.5 Å
R factor	0.317
R free	0.351
Number reflections used	30393
Total number of atoms	13194
Total number of amino acid residues	1764
Total number of bases	10

Crystallization statistics were calculated by Scala [CCP4, 1994] and SFCHECK [Vagin *et al*., 1999]. Refinement statistics were calculated by Refmac [Winn *et al*., 2001]. No Ramachandran outliers are present in the model.

Supplemental References

Callaghan AJ, Marcaida MJ, Stead JA, McDowall KJ, Scott WG, et al. (2005). Structure of Escherichia coli RNase E catalytic domain and implications for RNA turnover. Nature, 437, 1187-1191.

Campbell, J. L., Hopman, T. L., Maxwell, J. A. and Nejedly, Z. (2000) The Guelph PIXE software package III: Alternative proton database. Nuclear Instruments and Methods in Physics Research Section B: Beam Interactions with Materials and Atoms 170,193-204.

CCP4 (Collaborative Computational Project, Number 4) (1994). The CCP4 Suite: Programs for Protein Crystallography. Acta Cryst., D50, 760-763.

Grime, G. W. (2004) Dan32: recent developments in the Windows interface to GUPIX. In Tenth International Conference on Particle Induced X-ray Emission**,** (ed. M. Budnar), Portoroz, Slovenia.

Garman EF Grime GW (2005) Elemental analysis of proteins by microPIXE. Progress in Biophysics and Molecular Biology 89/2, 173-205.

Laskowski RA, MacArthur MW, Moss DS, Thornton JM (1993). PROCHECK: a program to check the stereochemical quality of protein structures. J. Appl. Cryst., 26, 283-291.

Leslie, AGW (1992). Recent changes to the MOSFLM package for processing film and image plate data. Joint CCP4 + ESF-EAMCB Newsletter on Protein Crystallography, No. 26.

Vagin A, Richelle J, Wodak S (1999). SFCHECK: a unified set of procedure for evaluating the quality of macromolecular structure-factor data and their agreement with atomic model. Acta Cryst., D55, 191-205.

Winn M, Isupov M, Murshudov GN (2001). Use of TLS parameters to model anisotropic displacements in macromolecular refinement. Acta Cryst., *D57*, 122-133.
